# Food Chemicals and Epigenetic Targets: An Epi Food Chemical Database

**DOI:** 10.1021/acsomega.4c03321

**Published:** 2024-05-29

**Authors:** K. Eurídice Juárez-Mercado, Juan F. Avellaneda-Tamayo, Hassan Villegas-Quintero, Ana L. Chávez-Hernández, Claudia Daniela López-López, José L. Medina-Franco

**Affiliations:** † DIFACQUIM Research Group. Department of Pharmacy, School of Chemistry, Universidad Nacional Autónoma de México, 04510 Ciudad de México, Mexico; ‡ PECEM, School of Medicine, Universidad Nacional Autónoma de México, 04510 Ciudad de México, Mexico

## Abstract

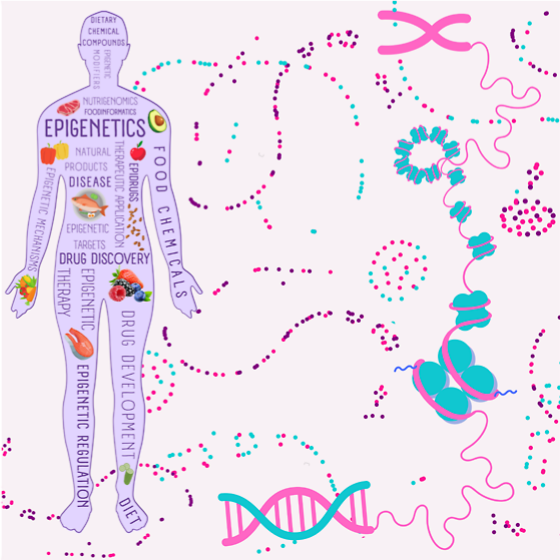

There is increasing awareness of epigenetics’s importance in understanding disease etiologies and developing novel therapeutics. An increasing number of publications in the past few years reflect the renewed interest in epigenetic processes and their relationship with food chemicals. However, there needs to be a recent study that accounts for the most recent advances in the area by associating the chemical structures of food and natural product components with their biological activity. Here, we analyze the status of food chemicals and their intersection with natural products in epigenetic research. Using chemoinformatics tools, we compared quantitatively the chemical contents, structural diversity, and coverage in the chemical space of food chemicals with reported epigenetic activity. As part of this work, we built and curated a compound database of food and natural product chemicals annotated with structural information, an epigenetic target activity profile, and the main source of the food chemical or natural product, among other relevant features. The compounds are cross-linked with identifiers from other major public databases such as FooDB and the collection of open natural products, COCONUT. The compound database, the “Epi Food Chemical Database”, is accessible in HTML and CSV formats at https://github.com/DIFACQUIM/Epi_food_Chemical_Database.

## 1 Introduction

The concept of epigenetics has changed since it was first introduced in the 1940s by Conrad Waddington to describe “the branch of biology which studies the causal interactions between genes and their products which bring the phenotype into being”.^1^ Nowadays, the meaning of epigenetics is accepted as the study of heritable changes in the gene expression profile that do not entail a change in the DNA sequence but modify the accessibility of the code via DNA methylation and modifications of amino acids on the amino-terminal tail of histones and noncoding RNAs.^1−3^ It has been proposed that these changes could be classified into three types: direct epigenetics, which occurs in the lifespan of a person; indirect epigenetics, which occur inside the womb due to events during gestation; and across indirect epigenetics, which refers to those changes that affected the individual predecessors and somehow, maybe through changes in the gametes or intrauterine environment setting, are transmitted across generations.^2^ The immense interest in the field led to many studies showing the link between epigenetic changes and certain diseases, such as diabetes, heart failure, cancer, inflammatory bowel diseases, and neurodegenerative diseases.^4−7^

**Figure 1:**
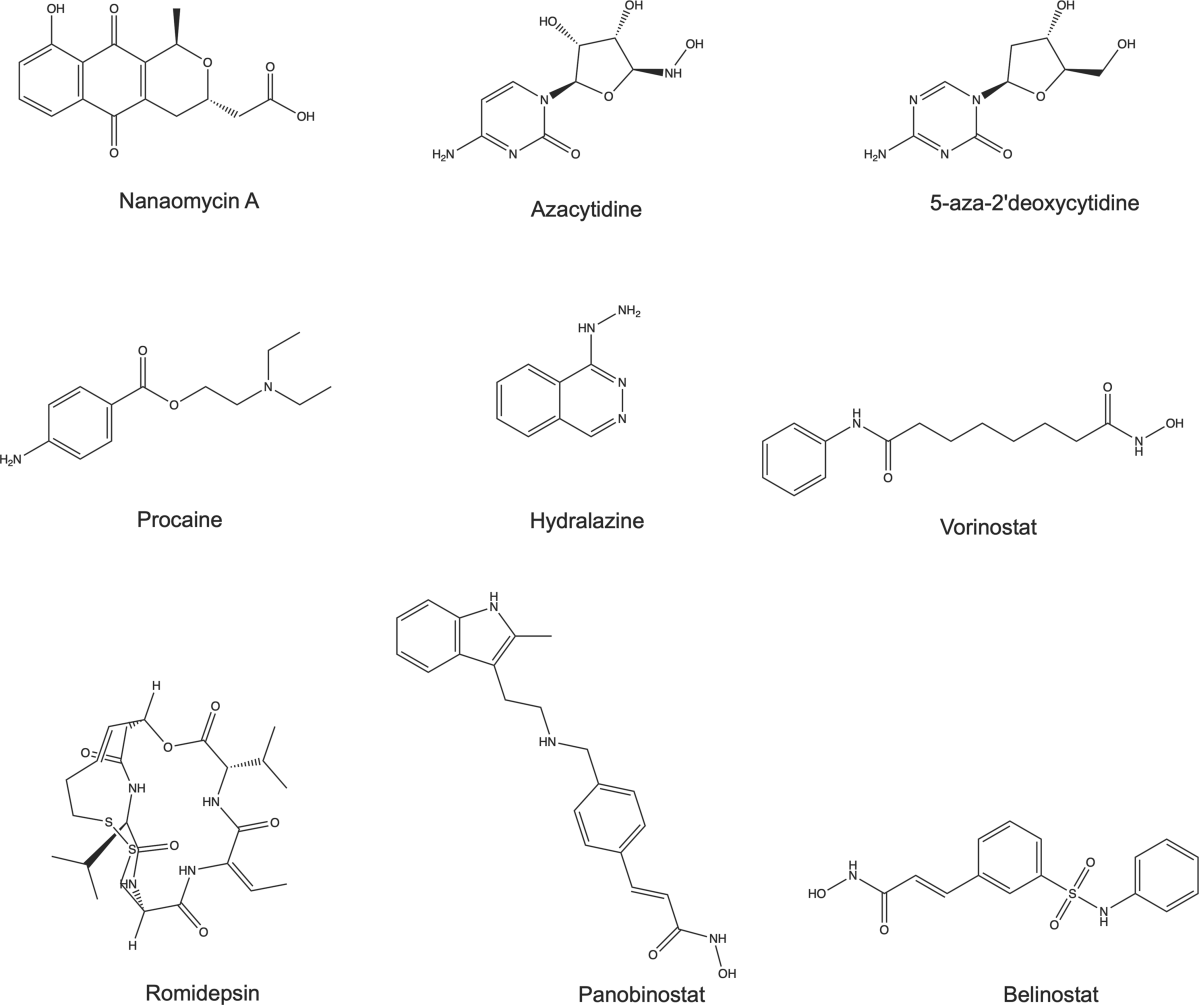
Chemical structures of representative epidrugs and epidrug candidates.

Certain enzymes have been described as having a key role in these epigenetic modifications: DNA methyltransferases (DNMTs), in charge of the covalent addition of a methyl group to the DNA, leading to the repression of certain genes; histone acetyltransferases (HATs), with the function of the acetylation of histone proteins, allowing the chromatin structure to open and become more transcriptionally active,^8^ and histone deacetylases (HDACs), which regulate the deacetylation of histones, leading to hypoacetylation toward heterochromatin and gene suppression.^9^ Thus, the search for molecules that could hit these targets began, and the term “epidrugs’’ was coined to describe chemical compounds that alter DNA and chromatin structure, promoting the disruption of transcriptional and post-transcriptional modifications by the inhibition of DNMTs and HDACs, mainly. As of 2022, several compounds have been approved by the Food and Drug Administration of the USA for clinical use, while other compounds are chemical probes. Examples of representative epidrugs and epidrug candidates include azacytidine (DNMT1 inhibitor), 5-aza-2′deoxycytidine (DNMTs and HDACs inhibitor), procaine (DNMTs inhibitor), hydralazine (DNMTs inhibitor), vorinostat (HDACs inhibitor), romidepsin (HDACs inhibitor), panobinostat (HDACs inhibitor), and belinostat (HDACs inhibitor). Nanaomycin A is a promising probe molecule (DNMT3b inhibitor).^10−14^ The chemical structures are shown in Figure 1.

One of the most promising areas of this search is the field of nutriepigenomics, which is focused on the study of the interaction between food nutrients and the genome through epigenetic mechanisms, modulating the overexpression or silencing of specific genes and metabolic responses.^15−17^ The interaction between nutrition, epigenetic targets, and the development of certain diseases such as type I and type II diabetes, inflammatory diseases, liver fibrosis, and cancer have been discussed in the past few years, leading to new alternatives to mitigate the damage or prevent such conditions.^4,5,9,15,18−21^

Using chemoinformatics to analyze natural products^22^ and food chemical data sets is becoming increasingly widespread. The term foodinformatics, coined in 2014,^23^ captures chemical information’s application to food science. Several studies focused on the contents and diversity of food chemicals have been published, yielding useful information to organize and mine chemical information associated with food chemicals, which, ultimately, is at the core of informatics applications in chemistry.^24^ Similarly, chemoinformatics has a growing interest in natural product research,^25^ giving rise to the subfield of natural product informatics.^22^ Notable examples of the applications of chemoinformatics to food chemistry and natural product research are the development of large compound databases such as FooDB^26^ and the Collection of Open Natural Products (COCONUT).^27,28^ Despite the increasing evidence of the effect of food and natural product chemicals on epigenetic targets, there needs to be a comprehensive survey of the effect of food molecules on different epigenetic targets rather than focusing on a specific disease or a specific epigenetic target family.

This study aimed to analyze the recent progress of research on food chemicals and food components acting with epigenetic targets, building a compound database that integrates information on the chemical structure of food chemicals and other natural products with the epigenetic activity profile. The scientific papers and compound database were analyzed using chemoinformatics, data mining, and visualization approaches to identify the most frequent epigenetic targets and related therapeutic areas associated with food chemicals reported so far, the food chemicals and other natural products most studied, and their epigenetic activity profile. The chemical structure contents, diversity, and coverage in the chemical space of the compounds in the molecular database were evaluated using quantitative methods and data visualization techniques. Since a compound data set’s chemical diversity and chemical space depend on the structure representation, we explored the chemical multiverse, e.g., chemical space generated with multiple structure representations.^29^ As part of the analysis, we explored the relationships between the chemical structures and the epigenetic activity profile using the structure–property landscapes concept.^30^

## 2 Methods

### 2.1 Literature Search and Analysis

We conducted a meta-analysis of the literature of research papers published between January 2017 and March 2023 in peer-reviewed journals with digital object identifier (DOI) numbers, documenting the research of food chemicals interacting with epigenetic targets with potential therapeutic applications or disease prevention. The literature search was done in PubMed^31^ and Web of Science Core Collection^32^ databases using the following search terms: (“epigenetics” AND “food chemical(s)”) OR (“epigenetics” AND “natural products”) OR (“epigenetics” AND “therapeutic application”) OR (“epigenetics” AND “disease”) OR (“epigenetics” AND “drug discovery”) OR (“epigenetics” AND “drug development”) OR (“epigenetic targets” OR “epigenetic therapy” OR “epigenetic mechanisms” OR “epigenetic regulation” OR “epigenetic modifiers” OR “epidrugs” OR “nutritional epigenetics” OR “nutrigenetics”). As part of the analysis, the dietary compounds were determined in the abstracts of the selected papers. Then, the most common therapeutic indications associated with these compounds were selected in the related papers. Additional analyses were performed after assembling and annotating a compound database described in Section 2.2.

### 2.2 Compound Database of Food and Natural Product Chemicals Annotated with Epigenetic Activity

Based on the literature search and analysis described in Section 2.1, a compound database herein termed “Epi Food Chemical Database” was assembled using Google Sheets. The chemical structures were represented using the linear notation simplified molecular-input line-entry system (SMILES).^33^ The compound database was annotated with the following information: compound name; the international chemical identifier (InChI); the hashed version of InChI (InChIKey);^34^ main food source; if available, link of the compound to the FooDB or COCONUT databases (using the corresponding identifiers in those public databases); reference to the peer-reviewed paper using the DOI number; and activity profile with the epigenetic targets for which the given compound has reported activity. To facilitate subsequent analysis and rapidly identify trends in the data, the activity profile was represented as a vector of “1”s and “0”s to indicate if the compound has or has not reported activity with a given epigenetic target, respectively.

### 2.3 Chemoinformatic Analysis of the Chemical Database

The content and diversity of the chemical structures of the 187 compounds in the Epi Food Chemical Database were analyzed under three main types of analysis: (a) scaffolds and chemical diversity using structural fingerprints and chemical scaffolds, (b) distribution in chemical space, and (c) descriptive structure–activity relationships based on the concept of activity, or more general, property landscapes.^30^ Each of the three types of analysis is described below.

#### 2.3.1 Chemical Content and Diversity Analysis

The scaffold content analysis was based on the definition of Bemis and Murcko,^35^ which considers a scaffold as the rings in a molecule and the connectors of them. The analysis was performed using in-house code in Python with the modules MurckoScaffold from the RDKit library. Also, the chemical structures of the compound database were analyzed using well-established protocols and broadly used to characterize or assess the chemical diversity, namely, scaffold contents and structural diversity, using four molecular fingerprints: Molecular ACCEs System (MACCS) Keys (166 bits); Extended Connectivity Fingerprints (ECFP) radius 2 and 3; and RDKit fingerprints. The similarity analysis was calculated using the Jaccard-Tanimoto index.^36^

#### 2.3.2 Visualization of the Chemical Space

To visualize the chemical space of the compounds in the Epi Food Chemical Database, we generated a t-distributed stochastic neighbor embedding (t-SNE). This technique involves nonlinearly reducing dimensions by creating Gaussian probability distributions across high-dimensional space and then utilizing them to enhance a Student t-distribution within a lower-dimensional space through optimization. The lower-dimensional space conserves pairwise similarities from the original higher-dimensional space, resulting in clustering within the embedding space without a notable loss of the structural information.^37,38^

#### 2.3.3 Structure-Epigenetic Activity Profile

**Figure 2:**
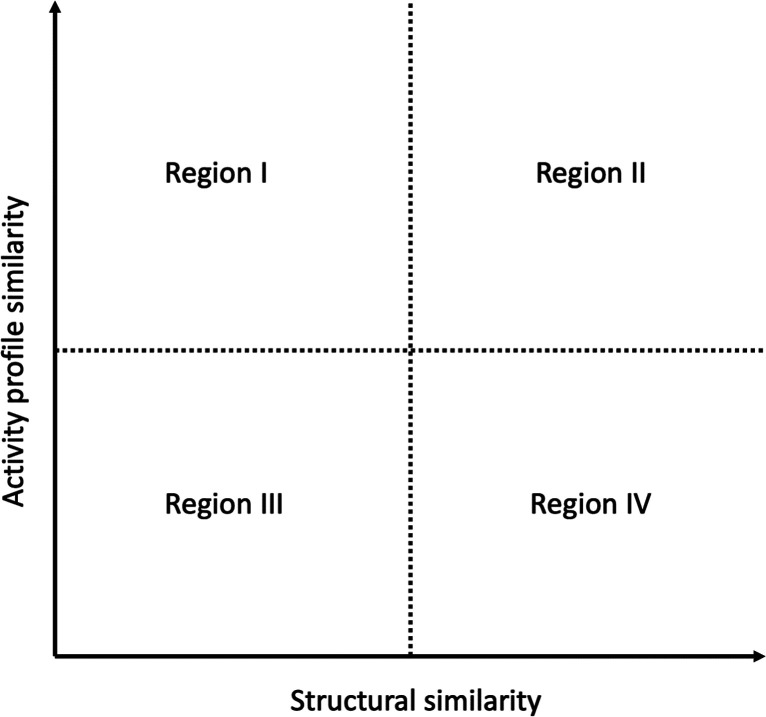
Prototype plot of a structure–activity similarity (SAS) map. Pairs of compounds in regions I and III have low structural similarity, while those in regions II and IV have high structural similarity. Pairs of compounds in regions I and II have a high similarity in their epigenetic activity profiles, although the chemical compounds in regions III and IV hold very different epigenetic activity profiles.

We computed all pairwise fingerprint-based and epigenetic activity profile similarities for the 187 Epi Food Chemical Database compounds. In both cases, we used the Jaccard-Tanimoto coefficient. The fingerprint-based similarity was calculated with four different fingerprints: ECFP4, ECFP6, MACCS Keys, and RDKit fingerprints.^39^ In total, 17,578 pairwise comparisons were computed for each fingerprint (including self-comparisons) and 17,391 pairwise comparisons for each fingerprint (excluding self-comparisons). The structure vs epigenetic activity profile similarity was plotted in a scatter plot reminiscent of the structure–activity similarity (SAS) maps.^40−43^Figure 2 shows a prototype plot of a SAS map where the epigenetic activity profile similarity is plotted on the *Y*-axis, while the fingerprint-based structural similarity is plotted on the *X*-axis. An SAS map can be roughly divided into four regions, as described in Figure 2; in Region I are pairs of compounds with very similar activity profiles but very different structural similarities. In Region II are pairs of compounds with high structural similarity and similar activity profiles. Region III identifies pairs of compounds with low structural similarity and very different activity profiles. In Region IV, there are pairs of compounds with high structure similarity but very different epigenetic activity profiles. It should be emphasized that the activity profile similarity computed in this work for each pair of compounds considers the bioactivity profile for all epigenetic targets, but the similarity value alone does not provide information regarding the specific set of epigenetic targets for which the compounds are active or inactive. However, the metric is useful in structure multitarget activity profiles.^34,42^

To select pairs of compounds with SAR activity correspondent to Region II of Figure 2, we identified those with a structural similarity greater than the average plus two standard deviations of the pairwise comparison distribution of the Epi Food Chemical Database, along with activity profile similarity value greater than 0.1. Conversely, for pairs of compounds in Region IV of Figure 2, also known as activity cliffs, we selected those with a structural similarity value greater than the average plus two standard deviations of the pairwise comparison distribution of the Epi Food Chemical Database and an activity profile similarity equal or less than 0.1.

## 3 Results and Discussion

### 3.1 Literature Analysis

The literature search revealed that the number of peer-reviewed papers found in PubMed and Web of Science using the search terms described in the Methods section was 7430 and 5960, respectively, of which 4484 were in both databases, 2946 were unique for PubMed, and 1476 were unique for Web of Science. Table 1 summarizes the major 20 types of diseases associated with epigenetics, and chemical compounds present in the food or natural products identified in the current search are listed. Table S1 in the Supporting Information summarizes the complete list associated with the respective related genes and epigenetic targets.

**Table 1:** Top 20 Types of Diseases Associated with Food Epigenetic Compounds

associated diseases	epigenetic target
breast cancer	DNMT1, DNMT3a, DNMT3b, HDAC1, HDAC2, HDAC3, HDAC 4, HDAC6, SIRT1, SIRT 2, SIRT 3, SIRT 4, SIRT5, SIRT6, KDM1B, KDM2A, KDM3A, KDM4A, KDM4B, KDM5A, KDM6B, KDM7A, KDM8
lung cancer	DNMT1, DNMT3a, HDAC4, HDAC5, HDAC6, HDAC8, HDAC9, SIRT2, KDM1A, KDM3B
prostate cancer	DNMT1, HDAC, HDAC4, HDAC5, HDAC6, KDM1A, KDM2B, SIRT1
colorectal cancer	DNMT, HDAC7, KDM6B
bladder cancer	HDAC6, LSD1, KDM6A
melanoma	HDAC2, HDAC5, KDM5A, KDM6A
oral cancer	HDAC6, HDAC8, KDM1A
hepatocellular carcinoma	DNMT3a, HDAC10, KDM1A, KDM2A
Alzheimer’s	DNMT, HDAC3, SIRT1
endometrial cancer	DNMT, DNMT1, HDAC 3, KDM4A
nonsmall cell lung cancer	DNMT3a, HDAC1, HDAC2, KDM6B
gastric cancer	DNMT1, DNMT3a, HDAC 2, KDM2A, KDM2B
cervical cancer	DNMT1, HAT/Ep300, HAT2B/Ep300, KDM5C
colon cancer	DNMT3b, HDAC 1, HDAC 3, HDAC 7, KDM4C, KDM5A, KDM6B
diabetes mellitus type 2	DNMT, HDAC, SIRT1
glioblastoma	LSD1, KDM1A
obesity and metabolic diseases	DNMT, HDAC1, SIRT1
esophageal carcinoma	DNMT, HAT2B/Ep300
squamous cell carcinoma	HDAC, HDAC5
atherosclerosis	DNMT, HDAC7, SIRT1

### 3.2 Compound Database

A total of 436 papers out of 8906 unique papers from both databases (PubMed and Web of Science) were used as the basis to build and curate the compound data set introduced in this work. The current data set version contains 187 unique compounds, of which 121 compounds have reported specific activity against at least one of the targets and 66 compounds have reported general activity. In this context, general activity refers to the reported activity of a compound against a family of epigenetic targets, where the specific target protein within that family was not precisely identified. The Epi Food Chemical Database contains ten columns with general information plus forty-nine columns that encode the epigenetic activity profile of the compounds across forty-six epigenetic targets. The general information is composed of structural data in three linear notations, namely, SMILES, InChi, and InChi keys, chemical name, source of the compound, DOI of the peer-reviewed reference reporting the epigenetic activity, and links to FooDB and COCONUT databases through hyperlinks using the corresponding IDs on these two public databases.

The epigenetic activity profile is encoded as bit vectors of 0 and 1, indicating the absence or presence of reported activity for each of the 46 targets (see Methods Section 2.2 for details). The epigenetic targets are ordered and arranged into three main groups: writers, erasers, and readers as follows: 8 writers (DNMT1, DNMT3a, DNMT3b, HAT/Ep300, HAT2B/Ep300, HAT3B/p300, EZH2, and PRMT1); 37 erasers (HDAC1, HDAC2, HDAC3, HDAC4, HDAC5, HDAC6, HDAC7, HDAC8, HDAC9, HDAC10, HDAC11, SIRT1, SIRT2, SIRT3, SIRT4, SIRT5, SIRT6, SIRT7, LSD1, KDM1A, KDM1B, KDM2A, KDM2B, KDM3A, KDM3B, KDM4A, KDM4B, KDM4C, KDM4D, KDM5A, KDM5B, KDM5C, KDM5D, KDM6A, KDM6B, KDM7A, and KDM8); and 1 reader (BET/BRD4). Epigenetic writers are responsible for selectively adding chemical modifications to histones or DNA, while erasers possess the capability to eliminate or erase chemical groups from histones and other proteins, thereby impacting chromatin structure and DNA accessibility. Reader proteins exhibit specificity in recognizing and binding to epigenetic marks, enabling the interpretation of chromatin information and the regulation of gene expression. Thus, they play an integral role in bridging epigenetic modifications to transcriptional machinery.^44^ The primary sources of the food chemicals in the Epi Food Chemical Database are meat, legumes, whole grains, grapes, poultry, acorn, acerola, strawberries, and nuts. Additional food sources are listed in the full Epi Food Chemical Database.

**Figure 3:**
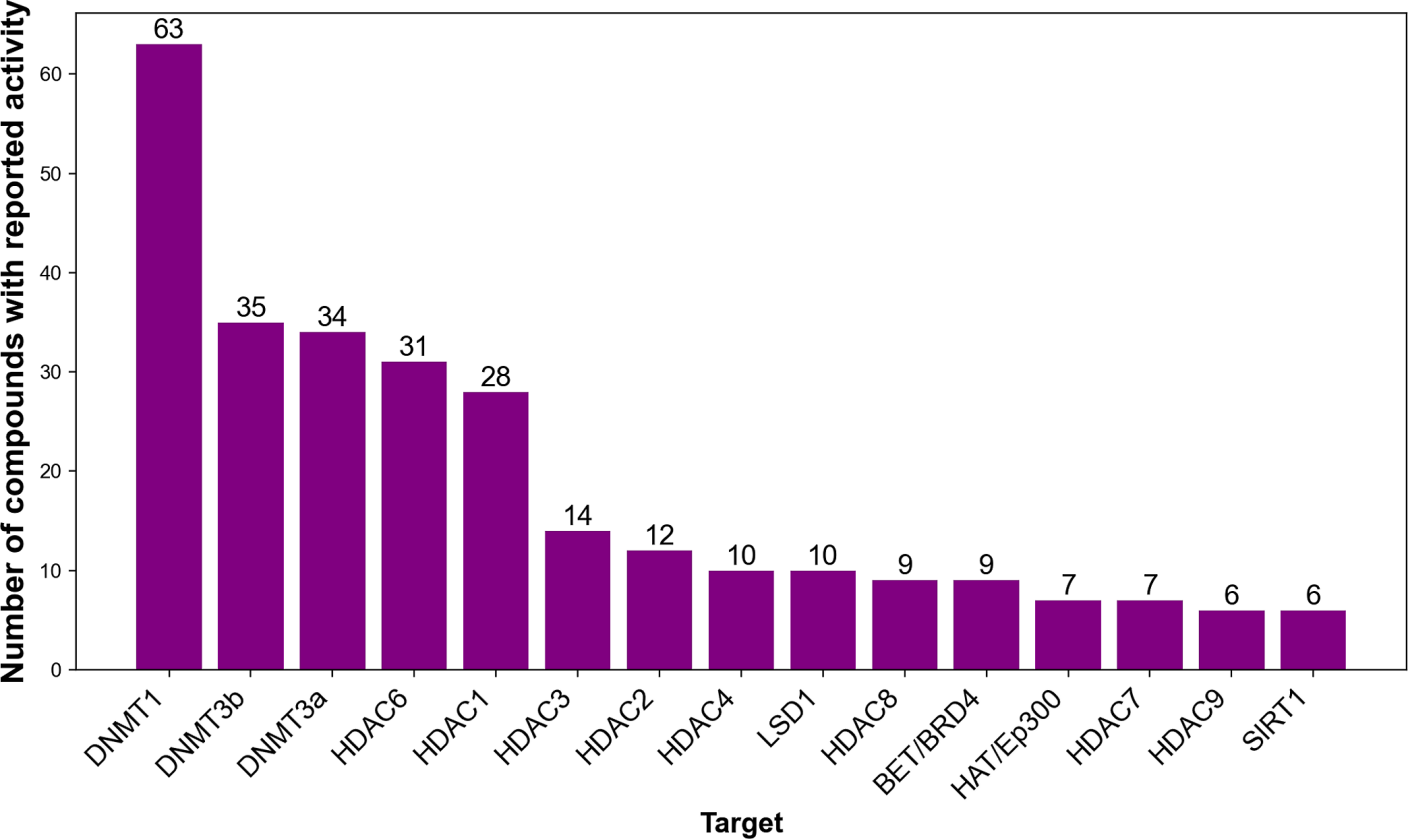
Histogram showing the 15 most frequent epigenetic targets.

The 15 most frequent targets with reported activity of the compounds in the database are shown in Figure 3. We can see that the most frequent target is DNMT1 (63), followed by DNMT3B (35) and DNMT3A (34), HDAC6 (31), and HDAC1 (28).

**Figure 4:**
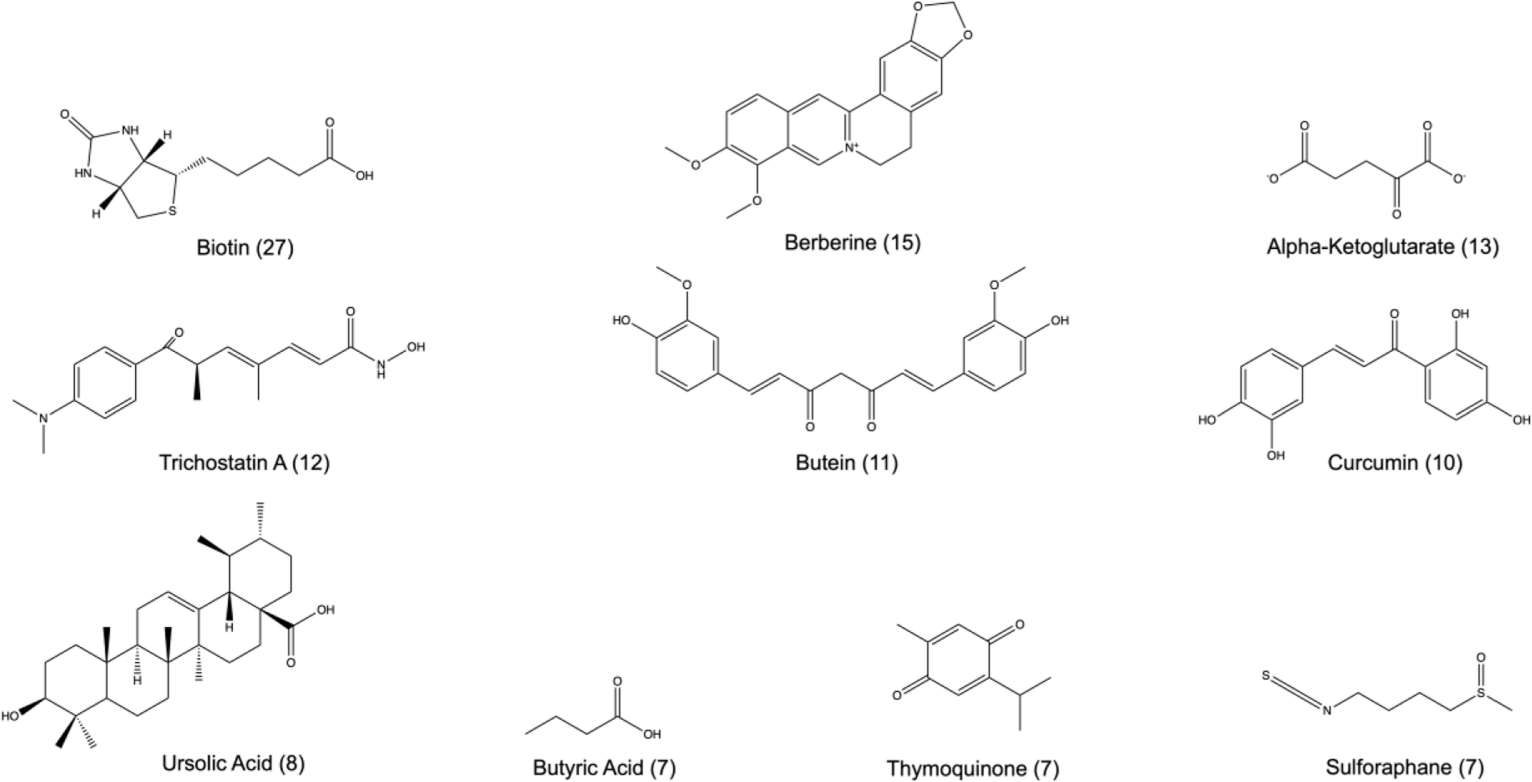
Top ten chemical compounds in the Epi Food Chemical Database with epigenetic activity.

There are 58 compounds with reported specific activity for only one target, being DNMT1 and HDAC6 the most frequent epigenetic targets with 18 compounds each, followed by LSD1 with eight compounds, BET/BRD4 with four compounds, and DNMT3a, DNMT3b, HAT/Ep300, and KDM4a with activity vs two compounds in any case. Furthermore, three epigenetic targets are associated with specific reported activity vs only one compound each: HDAC1 with phenethyl isothiocyanate (PEITC), SIRT1 with pterostilbene, and SIRT 5 with glutamate. The five compounds identified in the search with activity vs the largest number of epigenetic targets were: biotin (27 targets), berberine (15 targets), alpha-ketoglutarate (13 targets), trichostatin (12 targets), and butein (11 targets). These and additional compounds are shown in Figure 4, including the chemical structure and the number of targets in parentheses.

### 3.3 Chemoinformatic Analysis

#### 3.3.1 Diversity Analysis

**Figure 5:**
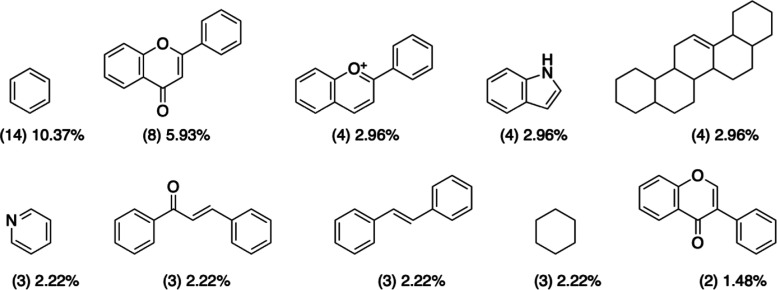
Ten most frequent scaffolds in the Epi Food Chemical Database.

The total number of unique scaffolds for the 187 compounds was 90. Figure 5 shows the ten most frequent chemical scaffolds, along with the frequency and percent proportion, which represent 35.54% of the total distribution. The most frequent scaffolds were benzene (10.37%), followed by flavone (5.93%) and flavylium (2.96%). Other frequent scaffolds were indole (2.96%), 1,2,3,4,4a,5,6,6a,6b,7,8,8a,9,10,11,12,12a,12b,13,14b-icosahydropicene (2.96%), pyridine (2.22%), (*E*)-chalcone (2.22%), (*E*)-1,2-diphenylethene (2.22%), cyclohexane (2.22%), and isoflavone (1.48%).

**Figure 6:**
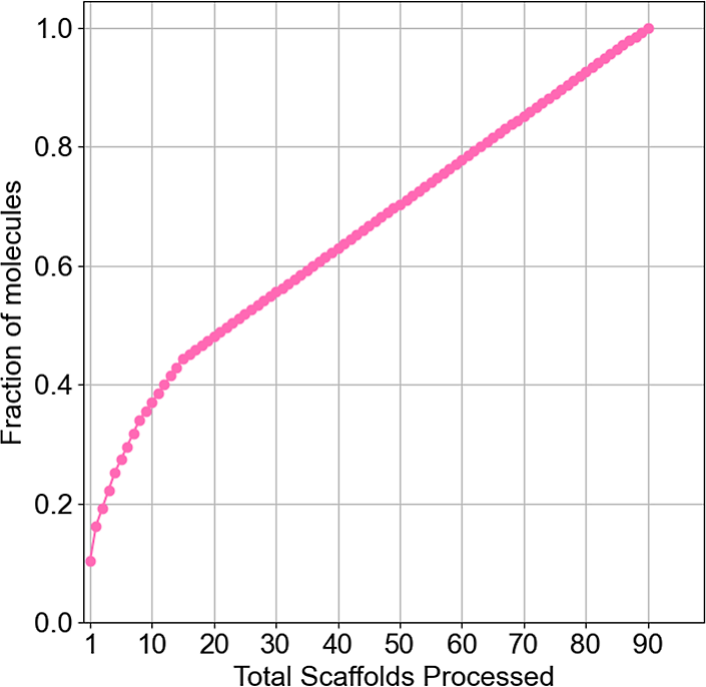
Cyclic system recovery curve of the Bemis and Murcko scaffold diversity.

Figure 6 shows the cyclic system recovery (CSR) curve for the scaffold diversity in the Epi Food Chemical Database. This curve illustrates the proportion of molecules within a data set that belong to a specific fraction of scaffolds. In a data set with high diversity, each molecule in the library would correspond to a different scaffold, resulting in a diagonal with an area under the curve (AUC) of 0.5. As the range of scaffold diversity diminishes, the curve will deviate from the diagonal orientation. Otherwise, the nadir of diversity would show in a data set wherein all compounds share the same chemical scaffold; in such an instance, the CSR curve would appear as a vertical line, accompanied by an AUC of 1.0.^45^ The shape of the CSR curve in Figure 6 indicates a large scaffold diversity of the Epi Food Chemical Database, with an AUC of 0.75. The large scaffold diversity is relevant because it suggests that there is broad variety of chemical structures in food chemicals that might regulate epigenetic mechanisms.

#### 3.3.2 Visualization of the Chemical Space

The chemical space of the Epi Food Chemical Database was visualized in a graphical t-SNE representation, with FooDB (52,856 compounds) included as a reference. t-SNE is a method for dimensionality reduction known for its ability to visualize high-dimensional data sets in spaces of two or three dimensions. It operates by calculating a similarity matrix among samples in the original space, typically employing measures such as Euclidean distance. Based on these similarities, the algorithm generates joint probability distributions in both the original and lower-dimensional spaces. The optimization process involves minimizing the Kullback–Leibler divergence between distributions, adjusting the position of points in the lower-dimensional space while preserving local structure, ensuring proximity between points that were closing neighbors in the high-dimensional space.^46^ Additionally, t-SNE’s capability to identify nonlinear patterns in data is essential for revealing complex structures that might go unnoticed with conventional methods.

**Figure 7:**
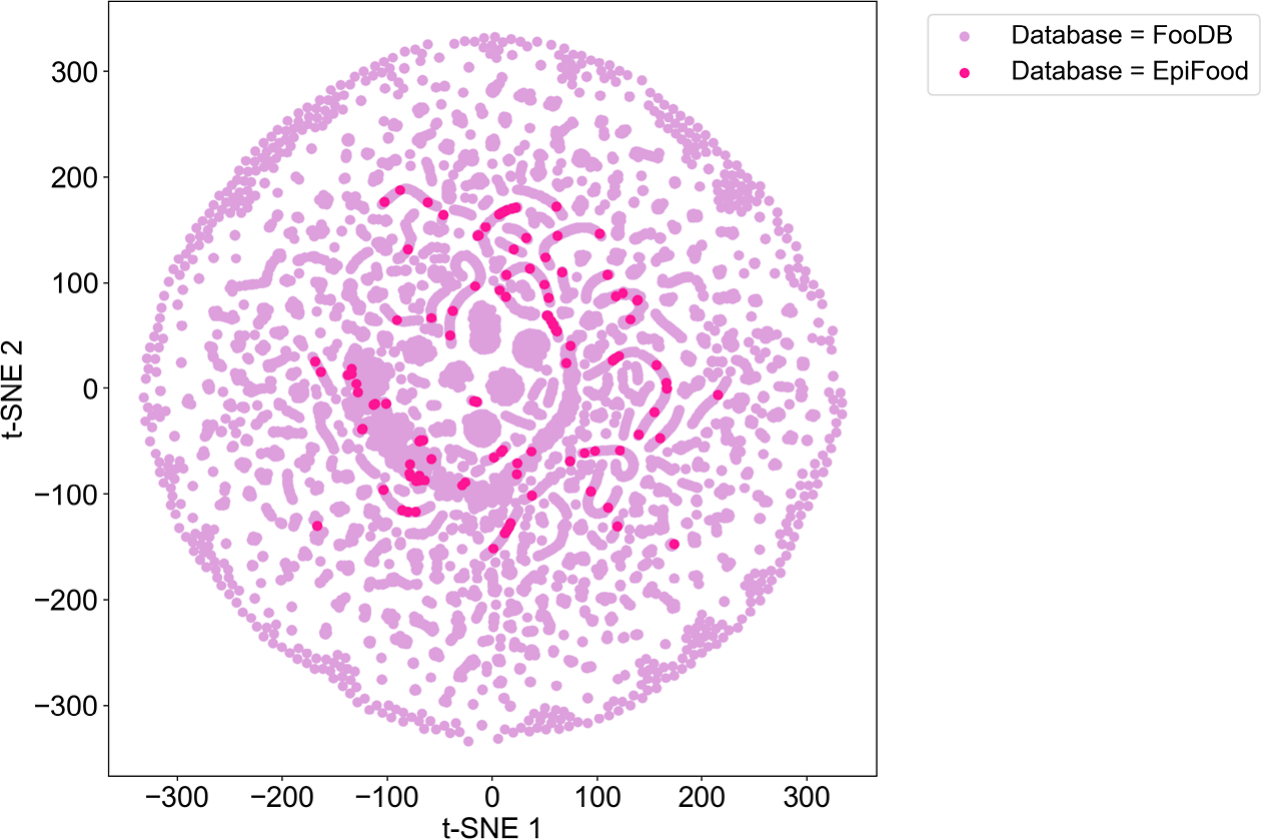
Visual representation of the Epi Food Chemical Database’s chemical space (deep pink) compared to the chemical space covered by FooDB (lilac).

The visualization using t-SNE on the chemical space of the Epi Food Chemical Database was performed based on the 209 descriptors in the module *MoleculeDescriptors* of RDKit. The descriptors include molecular weight, octanol/water coefficient (logP), number of hydrogen donor atoms (HBD), number of hydrogen acceptor atoms (HBA), topological polar surface area (TPSA), number of aromatic heterocycles, number of aromatic rings, number of heteroatoms, and the number of rotatable bonds. The visual representation of the chemical space shown in Figure 7 indicates the overall diversity of the newly developed database as compared to the space of the entire FooDB. A diverse chemical space allows for the representation of a wide range of chemical structure properties. Therefore, Figure 7 suggests that points representing chemical compounds in both databases share chemical space by occupying nearby regions in the two-dimensional space. The proximity of these points indicates similarities in the underlying calculated chemical properties of the represented compounds, suggesting common categories of chemical compounds or molecular profiles between the Epi Food Chemical Database and FooDB. Clusters of points denote compounds with similar properties.

FooDB was chosen as a reference for this analysis due to its status as the largest available food database to date, offering considerable breadth and representativeness in terms of the diversity of chemical compounds present in foods. It is important to note that the Epi Food Chemical Database was developed independently of FooDB. Therefore, it is relevant to consider that there are compounds in the Epi Food Chemical Database that are not found in FooDB. These compounds can be identified with the label “Not in database” in the corresponding column named “FooDB ID” within the Epi Food Chemical Database.

### 3.4 Structure-Epigenetic Target Activity Relationships

**Figure 8:**
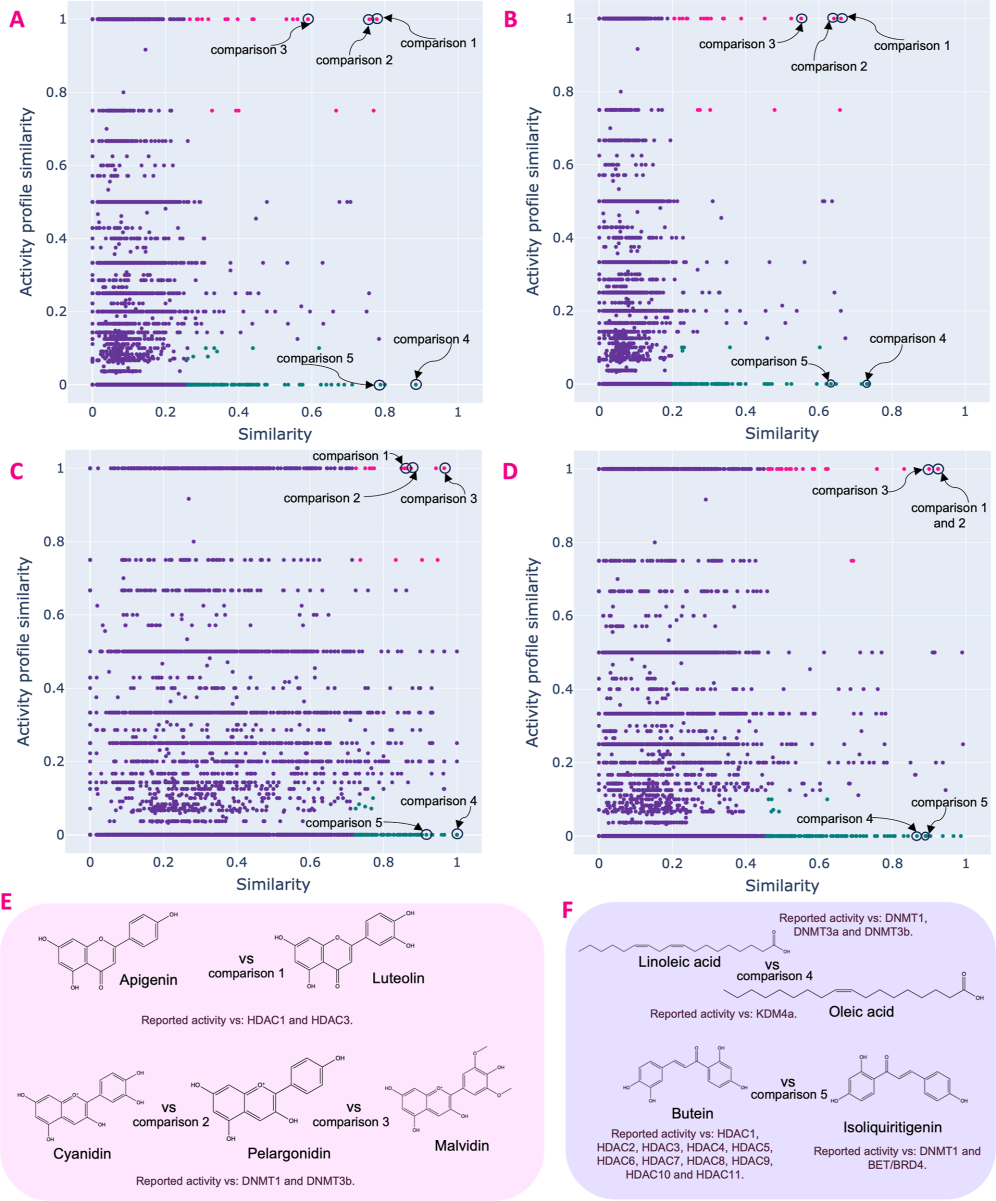
Structure activity-similarity (SAS) map of the Epi food Chemical Database. In pink are compound pairs in region II: similar structures and similar activity profiles; in green are compound pairs in the IV region: similar chemical structures but very different epigenetic activity profiles (activity cliffs). Maps generated with (A) ECFP4, (B) ECFP6, (C) MACCS Keys, (D) RDKit fingerprint, (E) examples of common compound pairs in region II (pink points) of all maps, and (F) examples of common compound pairs in region IV (green points) of all maps.

Figure 8 shows the SAS maps for the 187 chemical compounds in the Epi Food Chemical Database with the four different fingerprints: (A) ECFP4, (B) ECFP6, (C) MACCS Keys, and (D) RDKit fingerprint. The four interactive plots of the SAS maps are available in the Supporting Information in the html format.

The pink data points represent the pair of chemical compounds in region II of the SAS maps, which correspond to compounds very similar in structure as in profile activity. An example of this compound pair that is common in the SAS maps of the four fingerprints is apigenin vs luteolin (comparison 1 in Figure 8F). These compounds have reported activity vs HDAC1 and HDAC3, and some of the principal sources of both compounds are parsley, celery, onions, and pepper. Other examples of compounds in this region of the SAS maps are the comparisons between cyanidin vs pelargonidin vs malvidin (comparisons 2 and 3, respectively, in Figure 8); in this case, the compounds have reported activity vs DNMT1 and DNMT3b, and some of the principal common sources of the three compounds are blackberries, cherries, strawberries, and raspberries.

In contrast, the green data points in the SAS maps represent pairs of compounds in region IV, corresponding to compounds with similar chemical structures but very different activity profiles. Examples of these pairs of compounds present in region IV of all SAS maps for all the fingerprints are linoleic acid with reported activity vs DNMT1, DNMT3a, and DNMT3b and oleic acid with reported activity vs KDM4; their main sources are avocado, nuts, vegetable oils, and seeds. Another pair of compounds with very similar chemical structure but very different epigenetic activity profiles (Figure 8F) is butein with reported activity vs HDAC1, HDAC2, HDAC3, HDAC4, HDAC5, HDAC6, HDAC7, HDAC8, HDAC9, HDAC10, and HDAC 11, and isoliquiritigenin with reported activity vs DNMT1 and BET/BRD4, whose main sources are soybeans, peanuts, strawberries, and raspberries. It is important to emphasize that the pairwise epigenetic activity comparisons of the compounds in this work are based on the data published in the literature. For this reason, it is better to call them “pseudo activity cliffs” or pro-activity cliffs^34^ instead of activity cliffs for the compounds in region IV. This is because some pairs of compounds may have very similar activity profiles but have not been fully tested yet. Examples of these compounds are apigenin and luteolin vs chrysin. With current data reported in the literature, it is concluded that apigenin and luteolin are compounds that have similar structures with the same activity profile, with reported activity vs HDAC1 and HDAC3, but both compounds have pseudo activity cliffs vs chrysin, which has activity reported vs HDAC6. It is probable that chrysin could have activity vs HDAC1 and HDAC3 but also that apigenin and luteolin could also have activity vs HDAC6.

## 4 Conclusions

Herein, we report constructing and curating the Epi Food Chemical Database, which contains 187 chemical compounds from dietary and natural products. The database includes structural information and the epigenetic activity profile obtained from the literature vs 46 epigenetic targets. Breast cancer is by far the most discussed disease in the literature with the largest number of epigenetic targets that are dysregulated. We used chemoinformatic tools to compare and analyze the structural content, diversity, and chemical space. Scaffold analysis revealed that the most frequent scaffolds were benzene, followed by flavone and flavylium. Diversity analysis and coverage in the chemical space showed that the compounds in the Epi Food Chemical Database have an overall large diversity compared to compounds in FooDB. In addition, we identified two main groups of compounds: the first, with continuous structure–activity relationships, aka, fulfilling the similarity principle: compounds with similar chemical structures have similar epigenetic activity profiles. The second group of compounds can be considered pseudoactivity cliffs (similar structures but very different epigenetic activity profiles). This work serves as a justification for further experimental testing of the compounds that form pseudoactivity cliffs. They may have similar activities to their analogous compounds. This work contributes to the further advancement of a systematic analysis of food and natural product chemicals with epigenetic activity using chemoinformatic approaches.

## Data Availability

The Supporting Information is available at https://github.com/DIFACQUIM/Epi_food_Chemical_Database. It contains the annotated compound database of food chemicals reported with epigenetic activity (Epi Food Chemical Database) in HTML and CSV format; Table S1 with the list of diseases/genes obtained in the literature search; Table S2 summarizes the list of 436 research papers used to build the Epi Food Chemical Database and the interactive SAS maps plots of compounds in the Epi Food Chemical Database.
